# A solar radiation database for Chile

**DOI:** 10.1038/s41598-017-13761-x

**Published:** 2017-11-01

**Authors:** Alejandra Molina, Mark Falvey, Roberto Rondanelli

**Affiliations:** 1 University of Chile, Department of Geophysics, Santiago, Chile; 2 Center for Climate and Resilience Research, Santiago, Chile

## Abstract

Chile hosts some of the sunniest places on earth, which has led to a growing solar energy industry in recent years. However, the lack of high resolution measurements of solar irradiance becomes a critical obstacle for both financing and design of solar installations. Besides the Atacama Desert, Chile displays a large array of “solar climates” due to large latitude and altitude variations, and so provides a useful testbed for the development of solar irradiance maps. Here a new public database for surface solar irradiance over Chile is presented. This database includes hourly irradiance from 2004 to 2016 at 90 m horizontal resolution over continental Chile. Our results are based on global reanalysis data to force a radiative transfer model for clear sky solar irradiance and an empirical model based on geostationary satellite data for cloudy conditions. The results have been validated using 140 surface solar irradiance stations throughout the country. Model mean percentage error in hourly time series of global horizontal irradiance is only 0.73%, considering both clear and cloudy days. The simplicity and accuracy of the model over a wide range of solar conditions provides confidence that the model can be easily generalized to other regions of the world.

## Introduction

The main energy source for atmospheric and ocean dynamics is solar radiation. Of all the solar energy that reaches our planet, roughly 70% is absorbed (by the earth’s surface and the atmosphere) and the remaining energy is reflected to space. Nowadays, we are able to collect part of that energy and use it to produce heat and electricity. The development of new types of solar energy collectors (PV and CPS) not only makes this type of energy competitive with fossils fuels, but also has less negative environmental impacts, as solar energy is considered a renewable energy source and cleaner than fossil fuel energy production. Chile’s Atacama Desert has been shown to have the highest long term solar irradiance of any place on Earth^1^ and it is a national priority to encourage the use of this resource according to the National energy strategy 2012–2030^2^ and the National energy policy^3^. Many other physical and biophysical processes require knowledge of surface solar irradiance at high space and time resolution, such as the calculation of hydrological budget^4^, surface energy budget at the ocean^5^ and the growth of photosynthetic organisms over land and oceans. For these reasons, it is critical to have an accurate quantification of the solar energy at high temporal and spatial resolutions throughout the country.

Historical databases of the solar resource in Chile consist of both surface observations of horizontal global irradiance (GHI) and simulated data from atmospheric models^6–10^. The measurement based dataset consists of maps of interpolated global horizontal irradiance for all the national territory based on observations from the National Solarimetric Archive^6^. However, this database only considers 89 stations with measurements between 1961 and 1983, with many incomplete years and a lack of reliable metadata (information about the instruments and operational procedures to measure). For instance, the comparison of the database of the National Solarimetric Archive with the measurements of the National Energy Commission^11^, reveals differences of up to 20% in many areas^12^, that render this particular information unreliable for plant design.

Along with the previously mentioned national database, there are international databases that provide GHI and direct normal irradiance (DNI) which include data for the Chilean territory such as Meteonorm^8^, SolarGIS^9^, SSE-Nasa (Surface meteorology and Solar Energy)^10^ and the CERES Synoptic product^13^. The first two are paid-access databases and the latter two, SSE-Nasa database and CERES Synoptic product, have a relatively coarse spatial resolution (1 degree), which can lead to large errors in coastal areas (where large topographic and cloud distribution gradients can occur) or in regions with complex topography. The National Institute of Space Research (INPE) of Brazil has also developed GHI maps for all South America, using the GL1.2 model, which incorporates cloudiness information from the GOES EAST satellite^7,12^. These maps from INPE consist of 5-day GHI averages, with 0.4 degrees spatial resolution. Ortega *et al*.^12^ compared this data with national observations, and they found a mean percentage error (MPE) of 7.2% for the entire year and up to 40% during winter months. Such large errors are considered unacceptable for solar plant design purposes.

With the aim of reducing entry barriers to the solar industry, the Chilean government has promoted the development of a long-term, high spatial resolution and locally validated database of total solar irradiance at the earth’s surface. We present in this paper a public on-line, validated database of the solar energy resource for the entire Chilean territory (excluding Antarctic territories), calculated with a radiative transfer model and satellite data, with hourly data from 2004 until 2016 at 90 meters spatial resolution. The next sections describe in detail the methodology, the results and the validation approach.

## Methodology

For a given solar elevation, the amount of radiation that reaches the surface depends on the composition of the atmosphere along the path between the sun and the surface, of which the most variable component is the liquid and solid water within clouds. One possible approach for evaluating solar irradiance is using mesoscale numerical atmospheric models, that simultaneously predict the behavior of the radiatively active substances in the atmosphere and their impact on surface irradiance. However, accurate cloud representation is a difficult problem for numerical atmospheric models, especially for clouds that have small horizontal or vertical scales, such as convective cumulus and stratocumulus^14,15^. Many such models largely overestimate surface solar irradiance, which can be traced directly to an underestimation of cloud cover^4,16^. In addition, given the complexity of radiative transfer processes for cloudy conditions in numerical weather prediction models^17^, it would take significant computational time and resources to create such long-term database (with no guarantee of accuracy due to the cloud biases described above). As such, for the purpose of solar irradiance estimation of past conditions, it seems more appropriate to characterize cloudiness using observed fields from satellite, and many models have been formulated using this type of data to incorporate clouds in solar irradiance calculations^18–23^.

**Figure 1:**
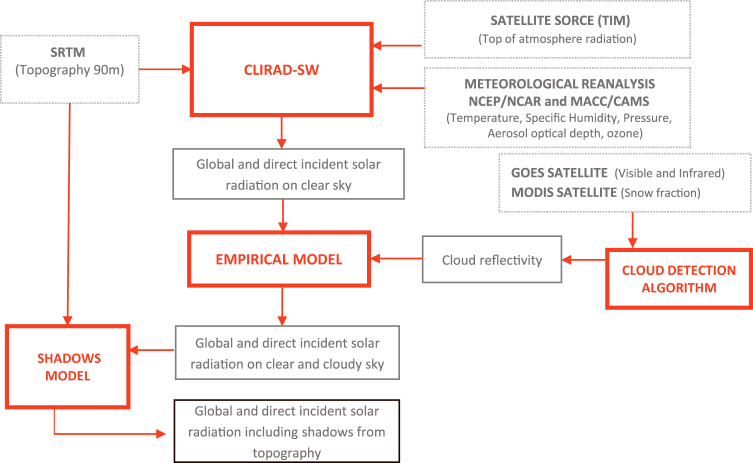
This scheme shows the flow of the methodology used to estimate GHI and DNI. Solid thick rectangles represents the models used. The doted rectangles show the input variables for each model. The solid thin rectangles show the output from the models.

In this section the methodology used for global horizontal irradiance (GHI), direct normal irradiance (DNI) and diffuse horizontal irradiance (DHI) over Chile is described. A schematic diagram of the methodology is presented in Fig. 1. First, the CLIRAD-SW radiative transfer model^24^ has been used to characterize the influence of gases and aerosols on the incident solar irradiance for clear sky conditions. Second, we apply statistical algorithms to detect cloud cover using the visible channel and the infrared channels 2 and 4 from the GOES EAST satellite. Third, for cloudy periods the radiation reflected by clouds is subtracted from clear sky irradiance to obtain a first approximation of the GHI and DNI at the surface. And finally, an empirical model has been derived for cloudy periods, which allows us to include the effect of absorption produced by clouds.

### Clear sky irradiance

#### Radiative transfer model

We use the CLIRAD-SW radiative transfer model to estimate the clear sky solar irradiance (DHI, DNI and GHI). CLIRAD-SW was developed by the NASA Laboratory for Atmospheres^24^ as a computationally efficient radiation scheme for use within general circulation models. It is a 1-dimensional model and requires the following input variables: the irradiance incident on the top of the atmosphere, vertical profiles of temperature, water vapor, liquid water, ice, aerosols and ozone, and total column amounts of carbon dioxide and methane. The model separates the solar electromagnetic radiation in eight spectral bands corresponding to the ultraviolet (bands 1 to 4), visible (band 5) and infrared (bands 6 to 8). Each one of these spectral bands interacts with different atmospheric components through absorption and scattering (for example, bands 1 to 5 absorb ozone, bands 5 to 8 absorb water vapor and band 8 absorb carbon dioxide).

CLIRAD-SW considers absorption from *CO*_2_, *O*_2_, *O*_3_ and water vapor using a k-distribution function^25^, where (*k*_*λ*_) indicates the absorption coefficient in the central *λ* wavelength for the corresponding spectral band. The absorber amount (*w*_*i*_) and the absorption coefficient in the vertical level *i* are scaled according to the pressure (*p*_*i*_) and potential temperature (*θ*_*i*_) of that level

1 $${w}_{i}({p}_{i},{\theta }_{i})={w}_{i}{(\frac{{p}_{i}}{{p}_{r}})}^{m}f({\theta }_{i},{\theta }_{r}),$$

2 $${k}_{{\lambda }_{i}}({p}_{i},{\theta }_{i})={k}_{{\lambda }_{i}}({p}_{r},{\theta }_{r}){(\frac{{p}_{i}}{{p}_{r}})}^{m}f({\theta }_{i},{\theta }_{r})$$

with this, the transmissivity of that level is calculated as *T*_*i*_(*w*):

3 $${T}_{i}(w)=\sum _{\lambda =1}^{n}{a}_{\lambda }({p}_{r},{\theta }_{r}){e}^{-{k}_{{\lambda }_{i}}{w}_{i}}$$

where, $f({\theta }_{i},{\theta }_{r})$ is a scaling function. For most molecules this function is negligible over the range of energy bands that affect total solar irradiance. The only significant scaling is associated with water vapor, in which the function is $1+\mathrm{0.00135(}{\theta }_{i}-{\theta }_{r})$. ${\theta }_{r}$ is a reference temperature of 240 K and ${p}_{r}$ a reference pressure equal to 300 hPa. The sum is over the *n* spectral bands that are considered to interact with each molecule and *m* is a constant coefficient, in this case, $m=0.8$.

Molecular scattering is parameterized as Rayleigh scattering, using constant coefficients that only depends on the corresponding spectral band. Incorporating aerosol effects on the model scattering requires additional inputs, such as the optical depth, the single scattering albedo and the asymmetry factor for each type of aerosol in each spectral band.

For each vertical level, the transmissivity and reflectivity are calculated for DNI and DHI using the *δ*-Eddington approximation^26^. Finally, radiative fluxes in each level are calculated, using the transmissivity and reflectivity values, through the adding two-stream fluxes approximation^27^.

Given DNI and DHI at surface level, GHI is calculated as

4 $$GHI=DNI\cdot \,\cos (Z)+DHI$$

where *Z* is the solar zenith angle for each particular time and location.

#### Meteorological inputs

Measurements of the incoming solar irradiance at the top of the atmosphere (TOA) made by the Total Irradiance Monitor (TIM) radiometer are used as input for the CLIRAD-SW model. TIM is an instrument on board SORCE, which is the most recent satellite launched by NASA to measure solar irradiance^28^. This data considers the annual variability of the TOA irradiance given by the eccentricity of earth’s orbit, solar activity and other types of variability such as the 11 year solar cycle. It is important to include this variability because it is linearly related to the irradiance at the surface.

CLIRAD-SW explicitly incorporates the effect of each of the atmospheric components that are provided as input. For instance, vertical profiles of temperature, pressure and specific humidity from NCEP-NCAR reanalysis^29^ have been used with a spatial resolution of 2.5 degrees. As CLIRAD-SW is only used to calculate clear sky irradiance, no liquid water or ice profiles are needed as input. The ozone column and aerosol optical depth are obtained from the Monitoring Atmospheric Composition and Climate (MACC)^30^ database from 2004 to 2012 and from Copernicus Atmosphere Monitoring Service database (CAMS) from 2012 to the present. Both models are part of the European Centre for Medium-Range Weather Forecasts (ECMWF) Integrated Forecasting System which uses them to generate gridded analyses of the chemical composition of the atmosphere^31^. These databases have spatial resolution of 0.25 degrees and for this work we use daily time resolution. As input for CLIRAD-SW aerosol optical depths for the 8 spectral bands of the model were calculated, using optical depth data at 469, 550, 670, 865 and 1240 nanometers provided by CAMS and MACC. For each band, the angstrom coefficient was calculated using the AOD of the two closest spectral lines from MACC or CAMS. This coefficient was then used to calculate the AOD for the center of each band used by the CLIRAD-SW model. The aerosol single scattering albedo and asymmetry factor were selected to represent a rural aerosol type (0.98 and 0.97 respectively) for all bands. For carbon dioxide a constant value of 400 ppm is used, independently of the location and time, this value has been chosen to represent the CO2 global average for the last decade.

### Cloud characterization

Clouds are detected using the data from GOES EAST satellite^32^, which takes images every half hour almost every day for South America. This satellite captures images in five spectral channels, one visible (0.55–0.75 *μ*m) and four infrared (3.8–4.0 *μ*m, 6.7–7.0 *μ*m, 10.2–11.2 *μ*m and 11.5–12.5 *μ*m). The raw satellite images are acquired from the CLASS database of NOAA (http://www.class.ncdc.noaa.gov). A postprocessing algorithm^33^ was applied to the raw images in order to calculate reflectivity fields from the visible band and temperature fields from the infrared bands. The algorithm corrects drifts of the instruments and changes in satellites.

**Figure 2:**
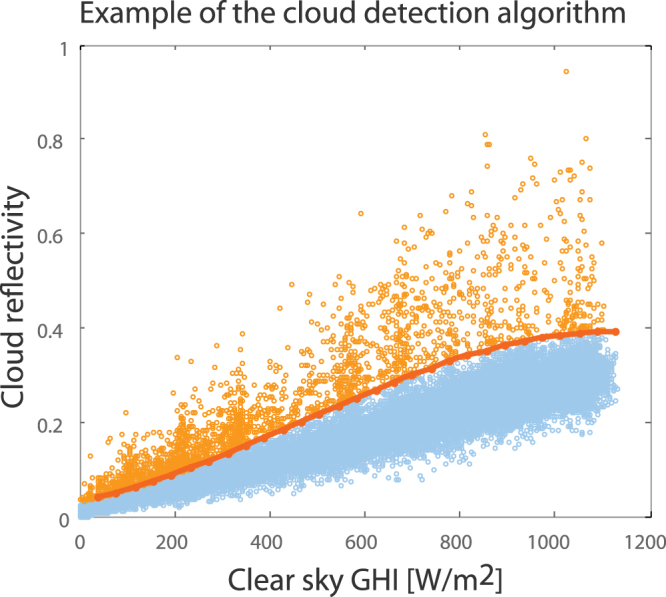
Example of the methodology of the cloud detection algorithm for a particular location. Each dot represents the satellite reflectivity with the corresponding clear sky irradiance. The orange solid line is the threshold set by the algorithm, the blue dots represent the clear sky and the orange dots the cloudy cases.

During daytime, solar visible irradiance is reflected either by clouds or the surface and detected by the satellite. When clouds are present, the reflectivity measured by the satellite usually exceeds the characteristic ground reflectivity. The present algorithm attempts to find a threshold that separates the reflectivity coming from the ground (clear sky case) from the one coming from a cloud (cloudy sky case). The reflectivity detected by the satellite is function of the solar irradiance incident on the reflective surface, so the threshold must also be a function of the incident solar irradiance. We developed a statistical method to separate clear from cloudy times for a single satellite pixel. For each pixel, the satellite albedo time series is related to the respective clear sky global horizontal irradiance at the corresponding time-steps. The clear sky cases cluster in the lowest range of satellite reflectivities. The cloudy cases are generally dispersed in the scatter plot with much higher reflectivities for a given irradiance (e.g. Fig. 2). To obtain the threshold between clear and cloudy cases, the satellite reflectivity data in a particular location are divided into 30 groups depending on the solar irradiance. For each group, a histogram is made with the reflectivity values, such that the average of the histogram data slope, divided by the histogram minimum value, is less than 0.1. Subsequently, the reflectivity threshold is chosen as the point in which the absolute value of the histogram slope is maximum. Of these 30 threshold points, we only select those that are in the range of the average of the 30 values plus/minus 1.5 times the standard deviation. A third degree polynomial function is then adjusted to points that meet the previous condition. This polynomial function is defined as the final threshold function used to distinguish between the reflectivity of the ground or cloud for any incident solar radiation.

For places where clouds are present over 40% of time, as in the Chilean Patagonia (latitudes southwards of 45° S), the algorithm is unable to find the threshold. For this area, a different algorithm was implemented. This alternative algorithm, based on Jedlovec^34^, uses infrared channels 2 and 4 (centered in 3900 nm and 10710 nm respectively) instead of the visible channel.

In this algorithm the brightness temperature is estimated from both channels in each pixel. The difference between the brightness temperatures of bands 4 and 2 is calculated (*D*(*t*)) and used to obtain the maximum positive difference (*D*_*pos*_) and the minimum negative difference (*D*_*neg*_) during the 20 previous days at the same time of day.

5 $${D}_{neg}=max(D({t}_{i-20}),\,\mathrm{...,}\,D({t}_{i-1}))\quad for\quad D({t}_{i}) < 0$$

6 $${D}_{pos}=min(D({t}_{i-20}),\,\mathrm{...,}\,D({t}_{i-1}))\quad for\quad D({t}_{i}) > 0$$

The value of the difference for a particular time-step is compared with the values of the previous step, and depending on the sign of the difference, the following conditions must be met for a time-step to be classified as cloudy:

7 $$If\,D(i) < 0\quad and\quad D({t}_{i})-{D}_{neg} < 5.1K$$

8 $$If\,D(i) > =0\quad and\quad D({t}_{i})-{D}_{pos} > 2.0K$$

Finally, a high clouds filter is applied. The channel 4 brightness temperature is compared with the maximum brightness temperature of the past 20 days at the same time of the day. If the temperature is at least 18 degrees lower, then that time is classified as cloudy.

The infrared cloud detection algorithm is only used when the visible algorithm fails. The reason for prioritizing the visible algorithm is that they have four times higher spatial resolution than the infrared ones.

### Empirical model for cloud attenuation

Clouds interact with incident solar radiation through scattering and absorption processes. Several studies^35,36^ have estimated the solar radiation absorbed and scattered by clouds using theoretical models and measurements campaigns. Using a radiative transfer model, Liou^35^ estimated that the amount of solar radiation absorbed by clouds reaches 9% for stratocummulus and cummulus, 15% for altostratus and altocummulus thin clouds (200 m) and could get up to 27% for clouds 5 km thick. To accurately calculate the amount of cloud scattering and absorption using a physical numerical model, would be necessary to know the entire vertical cloud profile (liquid/solid content and droplet size distribution) for each time and location. Currently, there is no such database with cloud profile information with the spatial and time resolution needed to calculate hourly irradiance. An alternative method is to use cloud reflectivity data from satellite images to infer the effect of the physical properties of the cloud on solar radiation through an empirical model.

In this work, we developed a methodology based on GOES satellite images to calculate an empirical function that relates the clear sky irradiance to the cloud reflectivity to estimate the effects of the cloud cover in the final irradiance.

Using the radiative transfer model, the clear sky solar irradiance (*GHI*_*cs*_) is calculated. In a first approximation, one can neglect the effect of cloud absorption on solar radiation and consider that surface irradiance *GHI*_*R*_ is just the clear sky radiation minus the radiation reflected by the clouds,

9 $$GH{I}_{R}=GH{I}_{cs}\cdot \mathrm{(1}-{A}_{V})$$

where, ${A}_{V}$ represent the reflectivity from the visible band of GOES for cloudy pixels.

**Figure 3:**
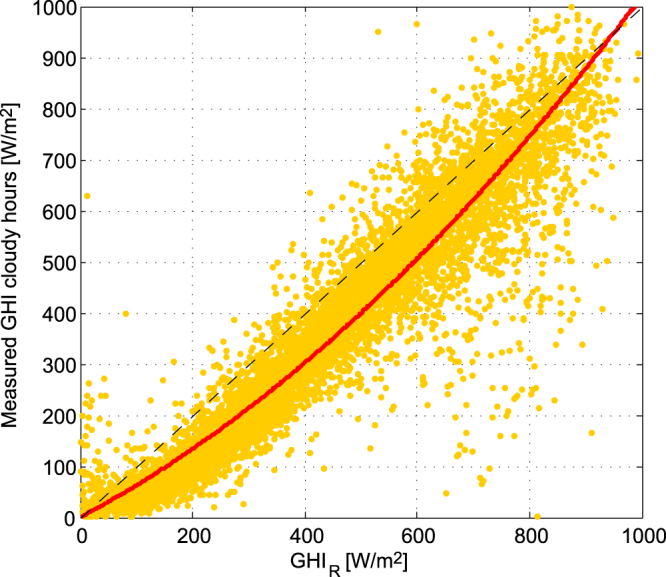
Fitted curve for the empirical model of GHI attenuation by clouds. The yellow dots are the irradiance under cloudy conditions calculated with the first approximation model (equation 8) versus measured irradiance for the same time and location under cloudy conditions. The red line correspond to the fitted quadratic function (equation 9).

The irradiance calculated considering the cloud reflectance effect only (*GHI*_*R*_), is compared against global horizontal irradiance measured at 40 ground stations for cloudy cases (see Fig. 3). Stations have been chosen in the central and southern regions of the country due to their high frequency of cloudiness. When the measured irradiance is smaller than about 850 $W/{m}^{2}$, equation 8 overestimates the irradiance under cloudy conditions, this can be explained by the missing effect of absorption by clouds. On the other hand, when measured irradiance exceeds 850 $W/{m}^{2}$, equation 8 underestimates it. This is caused by the overestimation of the cloud reflectivity in semitransparent clouds, as GOES satellite detects the total visible radiation from both clouds and terrain. To correct these effects, an empirical function (equation 9) is fitted to the data which relates solar irradiance under cloudy conditions (*GHI*_*cld*_) with *GHI*_*R*_.

10 $$GH{I}_{cld}=0.00044\cdot GH{I}_{R}^{2}+0.58\cdot GH{I}_{R}$$

Figure 3 shows that the empirical function provides a very good approximation to the observed irradiance over most of the range of *GHI*_*R*_, with a r-square of 0.96. Where the calculated irradiance exceeds 700 W/m2 there are several outliers where the observed irradiance is much smaller than the calculated irradiance. These points most likely correspond to cases where cloud reflectivity is underestimated due to incorrect illumination of cloud field by shadows of nearby clouds.

The same procedure is followed for the calculation of direct horizontal irradiance using measured DNI data from the Crucero II station from the Ministry of Energy network^11^. Direct horizontal irradiance (*DIR*_*cld*_) under a cloudy condition is calculated as

11 $$DI{R}_{R}=DI{R}_{cs}\cdot \mathrm{(1}-{A}_{V})$$

12 $$DI{R}_{cld}=0.0095\cdot DI{R}_{R}^{2}+-0.25\cdot DI{R}_{R}+15$$

Where *DIR*_*cs*_ is the clear sky direct horizontal irradiance. *DIR*_*cs*_ is calculated from the clear sky DNI data that results from the radiative transfer model, multiplying by the cosine of the zenith angle at each time. The same procedure has been applied to the DNI measurements to obtain measured direct horizontal irradiance (*DIR*_*cld*_).

Finally, DNI for cloudy conditions is calculated as direct horizontal irradiance for cloudy conditions divided by the cosine of the solar zenith angle at each time and location.

## Results

The GHI and DNI time series at one hour interval for the years 2004 until 2016 for a regular grid of 1 kilometer were estimated (the 1 km resolution corresponds to the approximate resolution of the GOES visible images). Additionally, shadows calculated with a 90 meter topography database have been applied to obtain a 90 meters solar irradiance database. The final database includes continental Chile, Easter Island and Robinson Crusoe island (these islands only have data from 2010).

With the GHI and DNI data it is possible to calculate diffuse horizontal irradiance (DHI) and global, direct and diffuse irradiance on tilted surfaces using a simplified method based on Perez^37^. Users can access the databases from the project website and have the possibility of download a file for a specific location with the entire simulated period any of the variables listed in Table 1. In addition, users can plot figures of the summarized information on the website, as the inter-annual variability of the GHI annual mean for an specific location, or download files with the typical meteorological year of several meteorological variables to be used with the standard software packages used for the energy generation calculation.

**Table 1:** List of variables in the database available in the Energy Ministry website.

Variable	unit
Global horizontal irradiance	W/m2
Direct horizontal irradiance	W/m2
Diffuse horizontal irradiance	W/m2
Global irradiance on a tilted surface	W/m2
Direct irradiance on a tilted surface	W/m2
Diffuse irradiance on a tilted surface	W/m2
Direct normal irradiance	W/m2
Daytime cloudiness	0–1
Fraction of the hour with shadows	%

### Results validation

**Figure 4:**
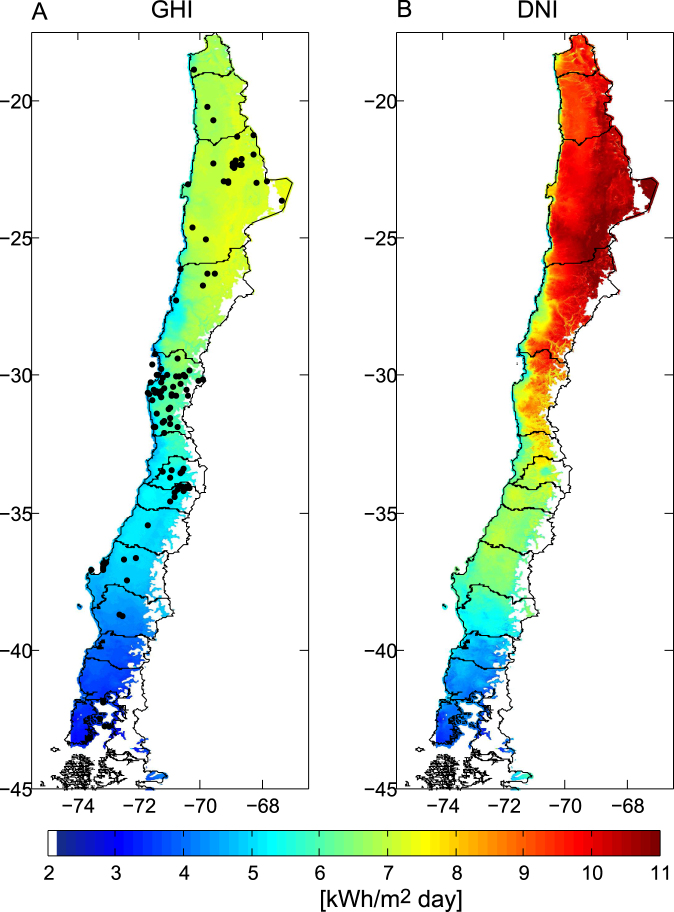
For each one kilometer grid point, the daily average insolation of GHI (panel A) and DNI (panel B), for the period 2004 to 2016, were calculated using the global horizontal and direct normal irradiance hourly time-series respectively (which were calculated with the methodology presented in this work). Black dots in panel A show the position of the ground stations used to validate the irradiance database. The calculation and plotting of the daily average insolation for each grid point were made using Matlab software 8.5.0.197613, Academic License number 1086178 (https://www.mathworks.com/).

Measured data of GHI have been collected from 140 stations belonging to CEAZA-MET, AGROMET-INIA, DMC, Chilean ministry of energy-GIZ and private companies. This station network covers a large part of the national territory, and captures the radiation response (and interaction) to all climates present in Chile. Figure 4 shows the location of the stations. Quality control of the data has been performed to classify the stations as high quality (67 stations) or low quality (73 stations). The data from high quality stations shows no statistical trends in hourly time series, no data obviously out of range and consistency between different years. These data can be used to validate the values of the solar irradiance database over different time scales. Low quality data have several types of errors, some of which have been corrected, but given our reduced confidence in these data they are only used to test the cloud detection algorithm and are not used to evaluate the irradiance estimates.

### Cloud detection validation

The cloudy cases are detected using the measured solar irradiance time-series. Cloudy periods in the observed data are identified when the measured irradiance deviates significantly from the theoretical clear-sky irradiance scaled according to each specific dataset, to account for trends in time instrumental bias. The steps to detect cloudy periods in measured GHI time series are:

1. Calculate clear sky GHI at the same location with the radiative transfer model.
2. Scale the clear sky GHI to eliminate bias between model and measurements in clear hours. To do this, a 1 degree polynomial has been adjusted for each day between the model and the measurements. A high pass filter has been adjusted to the coefficients of the polynomial for all days, to obtain the coefficients only on the clear days. Interpolating for all times, the coefficients are obtained for all times and applied to the clear sky GHI. This step is necessary for the measurements that have trends in time due to lack of maintenance or instrumental drift.
3. For a time to be classified as cloudy, it must meet any of the following criteria:

(a) The difference between measured and clear sky irradiance at the same time must be greater than 100 $W/{m}^{2}$.

(b) The difference between measured and clear sky irradiance at the same time must be greater than 50 $W/{m}^{2}$ and the slope between two consecutive times of the difference between measured and clear sky irradiance must be greater than 30 $W/{m}^{2}$.

(c) The difference of the length path of the two time series must be grater than 30, according to the criteria developed by Reno and Hansen^38^.

(d) The slope between two consecutive data is calculated for the measured and modeled GHI. The absolute difference between the two values must be greater than 30 $W/{m}^{2}$.

**Figure 5:**
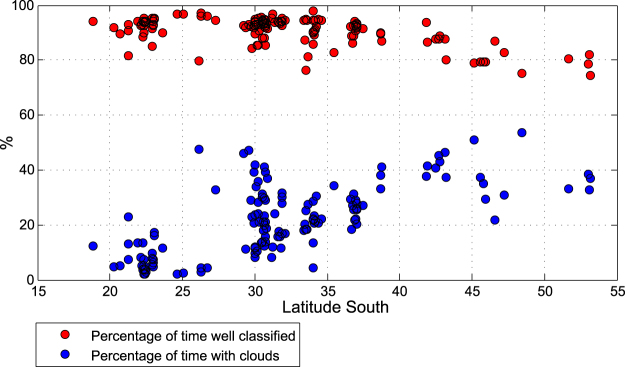
The red dots represent the percentage of time in which the algorithm correctly classified between clear or cloudy skies at each station. The blue dots represents the percentage of time with cloudiness according to the irradiance measurements. The stations are arranged from north to south (left to right).

The observed cloudy cases are compared against the simulated results. According to measurements, cloudiness in the north of the country (latitudes northern than 27° S) occurs less than 20% of the time, particularly in the Atacama Desert where cloud occurrence may be less than 5%. In the central region (between 27° S and 35° S) clouds are present between 20% and 30% of the time. However, south of 37° S cloudiness increases reaching values up to 60% along the western coast of Patagonia (blue dots in Fig. 5). The cloud detection algorithm matches the observation up to 95% of the time in the northern and central regions, but its accuracy decreases towards the south to around 80% in the frequently cloudy places (red dots in Fig. 5).

#### GHI validation

The high quality measured data have been used to validate the model. We calculated hourly mean time series with the measured GHI data and hourly time series of irradiance at the same locations using the methodology described in this work.

To compare measured and modeled time series, mean percentage errors and root mean square errors have been calculated for hourly and daily mean data.

13 $$MPE=\frac{1}{N}\sum _{i=1}^{N}\frac{{m}_{i}-{o}_{i}}{{o}_{i}}$$

14 $$RMSE=\sqrt{\frac{1}{N}\sum _{i=1}^{N}{({m}_{i}-{o}_{i})}^{2}}$$

where ${o}_{i}$ and ${m}_{i}$ are the individual values of hourly (or daily) mean measured and modeled respectively at one location. *N* is the number of hours or days in the time series.

**Figure 6:**
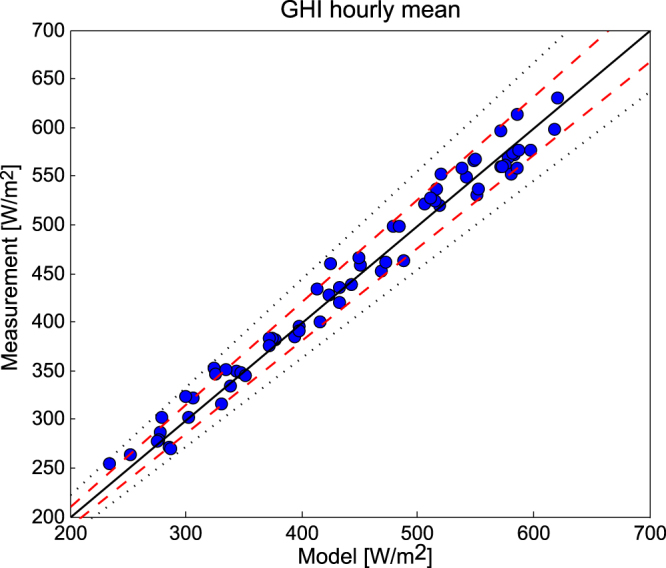
The dots represent the daily GHI average for the full period of measurements at each station (x-axis) versus the estimated GHI average for the same period. The segmented and dotted lines mark the difference ranges of 10% and 5% respectively.

**Figure 7:**
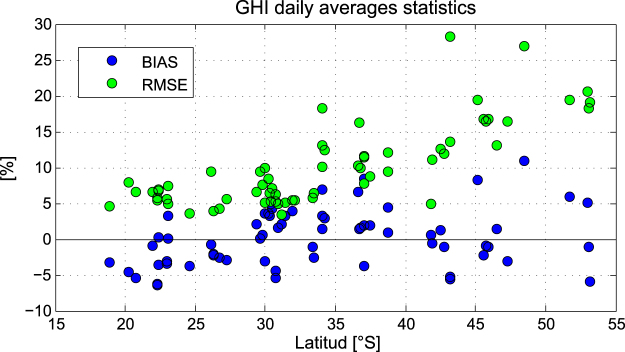
The blue and green dots shows the MPE (Mean Percentage Error) and RMSE respectively for the GHI daily average time series at each station.

The comparison of the modeled data and measurements is shown in Fig. 6, where each dot shows the MPE over the whole period at each location. The individual station MPE’s are all lower than 10% (see Fig. 7) and the RMSE is lower than 10% in northern stations and increases southward. Mean MPE and RMSE for all stations calculated with hourly time series and daily mean time series are presented in Table 2.

**Table 2:** Validation results of GHI.

Statistic	[%]
MPE clear sky hourly	0.13
MPE cloudy sky hourly	1.77
MPE total hourly	0.73
RMSE total hourly	20.81
MPE total daily	0.24
RMSE total daily	9.88

### Data availability

The datasets generated and analyzed during the current study are available free of charge from the “Explorador Solar” website www.minenergia.cl/exploradorsolar.

## Discussion and Conclusions

Cloudiness is the main factor that controls surface solar irradiance, so it is critical to have well defined distribution of cloudiness for the purpose of surface solar irradiance estimation. The cloud detection method presented has shown to work properly for most of cloud types (stratus, cumulus). However, five limitations should be carefully considered. First, during the sunrise and sunset, when the solar zenith angle is higher than 70 degrees, there might be large errors in cloud detection. Second, the algorithm has low sensitivity to cirrus clouds using the visible channel of the satellite. Third, clouds with a horizontal size less than a kilometer and clouds that last less than 30 minutes are also difficult to capture with present satellite data. Fourth, the geolocation of the satellite pixels for GOES 12 may have errors up to 4 kilometers. These errors potentially translate into large errors in surface radiance especially in regions of a sharp cloud distribution gradient such as the coastal region of Northern Chile. And lastly, over areas of permanent snow cover and salt lakes, in which surface reflectivities are similar or even higher than clouds, the calculation of irradiance is omitted. In addition to the problems noted above, some of the differences between modeled and measured cloudiness may be due to the fact that some of the clouds detected in the measured data could be really shadows of the environment. With no extra information, any decrease in measured irradiance has been used to infer the presence of a cloud, which may lead to an overestimation of observed cloudiness.

During periods of clear sky or in regions where clear sky are prevalent, aerosols give the greatest variability to the surface irradiance. The MACC and CAMS aerosol databases have not been fully validated for Chile, in part due to the lack of AOD measurements over the Desert, so we believe that a future validation and a better aerosol characterization could improve the performance of the model, particularly for DNI and DHI.

While global horizontal irradiance has been widely validated in Chile, the direct normal irradiance and diffuse horizontal irradiance should be used as a reference only, since they have only been validated with data from a single station (not shown in this paper), and the performance of the model for these two variables in the rest of the country is unknown.

The methodology used in this work was originally intended to be applied in the Chilean territory. However, given the accuracy of the model over the wide range of climates found in Chile, it is likely that the model can be generalized for the evaluation of solar irradiance in any part of the world.
